# Of energy and survival incognito: a relationship between viable but non-culturable cells formation and inorganic polyphosphate and formate metabolism in *Campylobacter jejuni*

**DOI:** 10.3389/fmicb.2013.00183

**Published:** 2013-07-09

**Authors:** Issmat I. Kassem, Kshipra Chandrashekhar, Gireesh Rajashekara

**Affiliations:** Food Animal Health Research Program, Ohio Agricultural Research and Development Center, Department of Veterinary Preventive Medicine, The Ohio State UniversityWooster, OH, USA

**Keywords:** viable but non-culturable cells, *Campylobacter jejuni*, inorganic polyphosphate, polyphosphate kinase, formate metabolism, formate dehydrogenase, acid stress, energy,

## Abstract

*Campylobacter jejuni* is a Gram-negative food-borne bacterium that can cause mild to serious diseases in humans. A variety of stress conditions including exposure to formic acid, a weak organic acid, can cause *C. jejuni* to form viable but non-culturable cells (VBNC), which was proposed as a potential survival mechanism. The inability to detect *C. jejuni* VBNC using standard culturing techniques may increase the risk of exposure to foods contaminated with this pathogen. However, little is known about the cellular mechanisms and triggers governing VBNC formation. Here, we discuss novel mechanisms that potentially affect VBNC formation in *C. jejuni* and emphasize the impact of formic acid on this process. Specifically, we highlight findings that show that impairing inorganic polyphosphate (poly-P) metabolism reduces the ability of *C. jejuni* to form VBNC in a medium containing formic acid. We also discuss the potential effect of poly-P and formate metabolism on energy homeostasis and cognate VBNC formation. The relationship between poly-P metabolism and VBNC formation under acid stress has only recently been identified and may represent a breakthrough in understanding this phenomenon and its impact on food safety.

## ENTER VBNC: A BRIEF HISTORY, SIGNIFICANCE, AND CONTROVERSY

Researchers in 1982 observed that two bacterial species, *Escherichia coli* and *Vibrio cholerae*, could not be retrieved from saltwater microcosms using a medium that previously sustained their growth (Xu et al., 1982). Although these bacterial species lost culturability in response to stress, they still maintained detectable metabolic activity, which suggested that these cells were still viable (Xu et al., 1982). Based on these observations, researchers suggested that stressed bacterial cells might exist in a viable but non-culturable (VBNC) state (Colwell et al., 1985; Oliver, 2010). Since the initial discovery, there have been hundreds of publications documenting VBNC formation in a variety of bacterial species, including important pathogens such as *Helicobacter pylori*, *V. cholerae*, *Campylobacter jejuni*, and others (Oliver, 2005). Further, it was shown that VBNC can be induced under different stresses, including exposure to chlorine, acids, oxygen, and pasteurization as well as those associated with fluctuations in the environment’s salinity, pH, and temperature (Chaveerach et al., 2003; Oliver, 2005). Today, VBNC are broadly defined as cells that enter a non-culturable state in response to stress, while maintaining a detectable but reduced metabolism (e.g., decrease in respiration, nutrient transportation, and synthesis of macromolecules), relatively high ATP levels, and aspects of cellular integrity such as intact chromosome content and cell membrane (Grégori et al., 2001; Oliver, 2005, 2010). Additional VBNC characteristics also occasionally include changes to cell morphology such as “rounding up” and reduction in the size of the cells, which is thought to maximize the surface area available for nutrient uptake while minimizing cell mass (Colwell, 2000; Oliver, 2005). It is important to note that the VBNC state may differ from other survival mechanisms. For example, in *Enterococcus faecalis*, the proteomic profiles of starved cells were observed to be different from those of the VBNC (Heim et al., 2002), which potentially indicated that the latter was triggered only in response to certain stresses.

With increasing knowledge about VBNC, their significance as a potential risk for public health became evident. A major concern is the inability to detect pathogens in the VBNC state using standard culture-based techniques. This is significant, because VBNC can potentially retain virulence and can be resuscitated back to “normal”/culturable physiological state under favorable conditions, including those available within hosts (Oliver, 2005, 2010). Subsequently, this may increase the potential for undetectable contamination and the spread of infectious agents to susceptible hosts. Although this is a contentious issue with arguments and research either supporting or disproving the ability of VBNC to cause disease in hosts, it is important to note that the possibility for infection should not be merely disregarded (Oliver, 2005, 2010). The lack of complete knowledge in regards to VBNC’s virulence, factors influencing their resuscitation, and cognate public health risks and ramifications might at the very least provide supportive impetus for prudence when considering risks associated with VBNC. In fact, under certain scenarios, potential risks may weigh heavily and can pose a severe threat to public welfare. For example, VBNC contamination of food products (Rowan, 2004; Dinu and Bach, 2011) and medical equipment (Zandri et al., 2012) can go undetected, impacting consumers, jeopardizing the safety of food, and threatening the lives of susceptible patients.

Of particular interest for food safety is *C. jejuni*, a food-borne pathogen that can form VBNC under stress (Rollins and Colwell, 1986; Jackson et al., 2009). *C. jejuni* is a Gram-negative bacterium that can cause disease in humans, including gastroenteritis and occasionally debilitating and life-threatening neuropathies (Vandamme and De Ley, 1991; On, 1996). The control of *C. jejuni* in poultry and other food animals and products (e.g., beef, turkey, and milk) has proved to be challenging, due in part to the atypical pathobiology of this bacterium, which lacks many of the classical stress response factors associated with other enteric pathogens (Parkhill et al., 2000). This singularity of *C. jejuni* necessitates a closer consideration of all its possible survival strategies, including VBNC formation; in order to enhance on-going efforts to reduce this pathogen in foods. This viewpoint is supported by research showing that *C. jejuni* VBNC can adhere to the skin of chicken carcasses (Jang et al., 2007), while a recent study reported that *C. jejuni* VBNC can still express a protein (CadF) that facilitates its attachment to host cells (Patrone et al., 2013). Further, it was also shown that *C. jejuni* VBNC can colonize suckling mice (Jones et al., 1991). Therefore, in this minireview, we will briefly discuss some of the molecular factors involved in VBNC formation in bacteria and focus in more detail on *C. jejuni*, highlighting recent research that associates specific metabolic pathways with VBNC formation in this important pathogen.

## UNTHREADING THE MYSTERY: GENETIC FACTORS INVOLVED IN VBNC FORMATION AND VIRULENCE

To date, many of the cellular triggers and genetic factors involved in VBNC formation are not well understood. However, increasing research into this phenomenon has revealed glimpses of potential factors that are likely involved in the persistence and expression of virulence in VBNC. Notably, it has been reported that gene expression in VBNC can continue for extended periods of time; for instance, the cytotoxin–hemolysin (*vvhA*) transcripts were detected in VBNC of *V. vulnificus* for up to 4.5 months (Saux et al., 2002). Although it is not clear if these expressed genes are directly involved in VBNC formation, the latter example highlights the possibility for maintaining virulence in the VBNC state. Further, many studies reported the expression of virulence-associated genes in VBNC of other pathogens. For example, in a recent study the expression of *cadF*, a gene that encodes an outer membrane protein that facilitates binding to fibronectin in host cells, was detected at high levels in *C. jejuni* VBNC for 3 weeks (Patrone et al., 2013). In parallel to these observations, the authors also reported that *C. jejuni* VBNC were capable of adhering to intestinal cells *in vitro*, but at levels that were lower than that of the culturable strain (Patrone et al., 2013). In another study, virulence-associated genes, including those encoding flagellin proteins, the cytolethal distending toxin, and a *Campylobacter* invasion antigen that are involved in invading- / interacting with the host’s intestinal cells were found to maintain a low level of expression in *C. jejuni* VBNC (Chaisowwong et al., 2012). Similarly, the mRNA of the Shiga toxin encoding gene (*stx1*) was detected in VBNC of *E. coli* O157:H7 (Yaron and Matthews, 2002), which is a notable finding because these toxins are associated with hemorrhagic colitis, hemolytic uremic syndrome, thrombocytopenia, hemolytic anemia, and renal failure (Karmali, 1989). Coccoid-shaped cells of *V. cholerae* entering a VBNC state were found to express the toxin co-regulated pilus (TCP), a virulence factor that is important for colonization of the small intestine in humans, and were able to colonize infant mice (Krebs and Taylor, 2011). The authors also noted that in a previous study TCP like appendages could be seen in micrographs of 1 year old *V. cholerae* VBNC (Chaiyanan et al., 2007). Another investigation detected viable-non-culturable and coccoid-shaped cells of *H. pylori* in biopsies collected from 12 dyspeptic patients, and these cells expressed *luxS*, a gene associated with quorum sensing and bacterial virulence (Cellini et al., 2008). Collectively, the aforementioned studies and other published research (**Table 1**) support the ability of VBNC to maintain some aspects of virulence and/or regain them after resuscitation.

**Table 1:** Example of studies that investigated the factors that triggerVBNC formation in *C. jejuni *and possible approaches to resuscitate these cells.

Reference	VBNC inducing factor(s)	Resuscitation	Expression of virulence genes	Other
Patrone et al. (2013)	Incubation in freshwater microcosms at 4°C	NA	*cadF* (mediates binding to fibronectin)	Adherence to human intestinal cells (Caco-2) *in vitro*
Chaisowwong et al. (2012)	Cold stress (4°C) in a nutrient rich medium (Bolton broth)	Co-incubation with Caco-2 in some experiments	Flagellar genes (*flaA*, *flaB*), *cadF*, *Campylobacter* invasion antigen gene (*ciaB*), cytolethal distending toxin genes (*cdtA*, *cdtB*, and *cdtC*)	Invasion of Caco-2 cells
Gangaiah et al. (2010)	Formic acid in Mueller-Hinton broth at 42°C	NA	NA	NA
Klancnik et al. (2009)	Short-term starvation (5 h incubation in a low nutrient medium)	NA^1^	NA	*In vivo* systemic campylobacteriosis in mice^1^. Adhesion, invasion, and survival in Caco-2 for up to 4 days^1^. Heat-stress resistance (55°C for 3 min)^1^
Gangaiah et al. (2009)	Formic acid in Mueller-Hinton broth at 42°C	NA	NA	NA
Guillou et al. (2008)	Storage in bottled water at 4°C in the dark	Inoculation into chicken embryonated eggs	NA	NA
Jang et al. (2007)	Aerobic conditions at 4, 25, and 37°C	NA	NA	Found after rinsing on artificially inoculated crevices and feather follicles of chicken skin^2^
Tangwatcharin et al. (2006)	Cold (4°C in *Brucella* broth) and heat-stress (60°C in brain heart infusion broth)	NA	NA	Some loss in the outer membranes of aging cell suspensions
Baffone et al. (2006)	Artificial sea water at 4°C	*In vivo* passage in the mouse intestine (dependent on the titer of respiring bacteria in the VBNC state; >10^4^ cell/ml)	NA	Colonization of mice
Ziprin and Harvey (2004)	Sterile water at room temperature	Failure to resuscitate in day-of-hatch leghorn and broiler chicks with experimentally introduced normal gut microflora	NA	No colonization of the chicken ceca 7 days post-VBNC inoculation
Ziprin et al. (2003)	Sterile water at room temperature	Failure to resuscitate in day-of-hatch leghorn chickens 1 and 2 weeks after inoculation	NA	No colonization of the chicken ceca
Chaveerach et al. (2003)	Mueller-Hinton broth with formic acid (pH = 4.0)	Inoculation into specific-free-pathogen fertilized chicken eggs	NA	Colonization of embryonated eggs
Thomas et al. (2002)	Simulated aquatic conditions at 10°C	NA	NA	NA
Cappelier et al. (1999)	Starvation in sterilized surface water (pH = 6.0) at 4°C	Inoculation into yolk sacs of embryonated eggs	NA	Ability to adhere to HeLa cells after resuscitation
Tholozan et al. (1999)	Starvation in sterilized surface water (pH = 6.0) at 4°C	NA	NA	Increase in VBNC cell volume, decrease in internal potassium content and the membrane potential. Only AMP was detected after 30 days of incubation
Lazaro et al. (1999)	Suspension in phosphate-buffered saline (pH = 7.3) in the dark at 4 or 20°C	NA	NA	Up to 7 months of viability. Intact chromosomal DNA (after 116 and 61 days at 4 and 20°C, respectively). Bleb-like membrane vesicles around cells at 4°C
van de Giessen et al. (1996)	Suspension in sterilized surface water and potassium phosphate buffer at 4°C	Failure to resuscitate in chickens and mice	NA	No colonization of the ceca and intestines of the chickens and mice
Stern et al. (1994)	Suspension in phosphate-buffered saline (pH = 7.2) at 4°C	Resuscitation in 2 out of 39 one-day old chickens	NA	Colonization of the ceca of some chickens
Medema et al. (1992)	Starvation in filter-sterilized and pasteurized surface water	Failure to resuscitate in 1-day old chickens and the allantoic fluid of embryonated eggs	NA	No colonization of the chicken ceca
Jones et al. (1991)	Sterilized pond water at 4°C	*In vivo* passage in suckling mice (only two strains out of four were retrieved)	NA	NA

*Information about virulence properties and gene expression in the VBNC is also highlighted. ^*1,2*^It was not clear if the authors in these studies used a culture/suspension that only contained VBNC cells.NA, not applicable.*

It was shown that two regulatory genes (*algU* and *gacA*) that code for the alternative sigma factor (σ^E^) and a response regulator, respectively, may be involved in VBNC formation in *Pseudomonas fluorescens* CHA0, which is used as a biocontrol agent against black root rot (Mascher et al., 2002). Additionally, it was suggested that resuscitation-promoting factor (Rpf)-like proteins might be involved in the reactivation of non-culturable cells of the human pathogen *Mycobacterium tuberculosis* (Shleeva et al., 2002). A delay in VBNC formation in an *S. *Typhimurium LT2 mutant was associated with a 99-bp in-frame deletion in the *clpX* gene, which is known to be involved in forming a protease complex that degrades the general stress sigma factor RpoS (Kusumoto et al., 2013). Subsequently, the authors suggested that this ClpX–RpoS relationship might have affected entry into the VBNC state (Kusumoto et al., 2013). Further, RpoS expression was detected for up to 14 days in VBNC of *V. vulnificus* (Smith and Oliver, 2006), while this stress factor was implicated in the persistence of *E. coli* in a VBNC state (Boaretti et al., 2003). In another study, the inactivation of OxyR, an oxygen stress regulator, and the cognate catalase enzyme impacted VBNC formation in *V. vulnificus* (Kong et al., 2004). Collectively, these are very interesting findings and can potentially shed light on the VBNC state of important pathogens and beneficial bacteria; however, this also raises several important questions. For example, the atypical pathogen, *C. jejuni*, has substantially documented VBNC state but lacks RpoS, OxyR, and a σ^E^ response (van Vliet et al., 1999; Parkhill et al., 2000), while investigations of a potential *C. jejuni* resuscitation factor (Cj0645) in strain NCTC11168 showed that the target was not an Rpf ortholog (Morgan, 2010). These observations suggest that the aforementioned genes may not necessarily be a factor in all VBNC-forming pathogens, which raises the following question: could there be a ubiquitously distributed cellular mechanism that might affect VBNC formation across many species? This question can perhaps be partially addressed by recent findings (detailed below) that link VBNC formation in *C. jejuni* to the metabolism of inorganic polyphosphate (poly-P), an ancient molecule that is ubiquitous in bacteria and plays a role in energy storage and production (Kornberg et al., 1999; Rao et al., 2009).

Despite the current gaps in knowledge, the studies highlighted previously present a convincing case for researching the virulence of VBNC and their potential impact on public health. This might be of critical relevance when considering the survival mechanisms of atypical pathogens such as *C. jejuni* and cognate ramifications to public health, including food safety.

## OF ENERGY AND VBNC: A RELATIONSHIP BETWEEN *C. jejuni* VBNC FORMATION AND INORGANIC POLYPHOSPHATE METABOLISM

Most of the past work that focused on *C. jejuni* VBNC mainly described the physical, chemical, and environmental triggers that induce this state such as exposure to oxygen, persistence in aquatic microcosms, changes in temperature and pH, and starvation (Jackson et al., 2009; **Table 1**). In addition, there was an emphasis on strategies aiming at resuscitation of *C. jejuni* VBNC *in vitro* or *in vivo* (Bovill and Mackey, 1997; Cappelier et al., 1999; Chaveerach et al., 2003; Baffone et al., 2006; **Table 1**). Like in many VBNC-forming bacteria, the genetic mechanisms that are associated with the VBNC state of *C. jejuni* are largely unknown. However, it was recently shown that VBNC formation in *C. jejuni* might be impacted by proteins involved in the metabolism of inorganic poly-P. Specifically, poly-P is a linear polymer of orthophosphate residues that plays a vital role in *C. jejuni* and other bacteria as a source of ATP for approximately 500 cellular reactions and as a modulator of stress and survival phenotypes (Kornberg et al., 1999; Rao et al., 2009; Kassem and Rajashekara, 2011). Since (1) maintaining cellular respiration and relatively high ATP levels are two major features of the VBNC state, (2) conserving energy is a basic survival strategy under stress, and (3) *C. jejuni*, a bacterium with relatively small genome that lacks typical stress responses, has invested in retaining a network of enzymes associated with poly-P metabolism, a link between the poly-P molecule and *C. jejuni* VBNC appeared to be plausible. The latter mandated a closer look at *C. jejuni* that revealed that this pathogen possessed two major enzymes, namely polyphosphate kinase 1 (PPK1) and polyphosphate kinase 2 (PPK2), which are principally involved in the synthesis/accumulation of poly-P and associated GTP production, respectively (Gangaiah et al., 2009, 2010). The inactivation of these enzymes leads to pleiotropic effects, influencing different survival phenotypes in *C. jejuni* (Gangaiah et al., 2009, 2010). Notably, live/dead cell staining analysis showed that the *C. jejuni* deletion mutants, Δ*ppk1* and Δ*ppk2*, possessed a significantly reduced ability to form VBNC after challenge with formic acid (Gangaiah et al., 2009, 2010). This was confirmed using flow cytometry analysis that revealed a significant change in the cell size and granularity of the Δ*ppk1* mutant as compared to the parental strain (Gangaiah et al., 2009), possibly indicating an increase in dead cells in the acid-stressed mutants (Kusters et al., 1997). While investigating the expression of a number of genes that were believed to contribute to the phenotypes of the Δ*ppk1* mutant, it was found that the phosphate regulon genes (*phosR*, *pstS*, *pstC*, and the periplasmic substrate binding protein-encoding gene, CJJ81176_0750), the multidrug resistance efflux pump gene (*cmeC*), the global post-transcriptional regulator (*csrA*), and the stringent response regulator (*spoT*) were not affected in the acid-stressed mutant cells (Gangaiah et al., 2009). However, *ppk2* was significantly down regulated in the formic acid challenged Δ*ppk1* mutant, but the expression of *ppk2* was not affected in similarly treated wild-type cells, which further implicates the *ppks* in *C. jejuni*’s VBNC formation (Gangaiah et al., 2009). Since the Δ*ppk1* mutant was deficient in the accumulation of poly-P and the *ppk2* down regulation would possibly reduce the associated GTP production (Gangaiah et al., 2009), the aforementioned observations suggest that the poly-P-dependent ATP/GTP pools and ratios in the *ppk* mutants might be deficient as compared to that of the parental strain. This assumption is supported by the lower levels of poly-P-dependent GTP and the higher ATP:GTP ratios that were detected in the Δ*ppk2* cells using thin layer chromatography analysis (Gangaiah et al., 2010). Therefore, it appears that the disruption of the poly-P associated enzymes creates an imbalance in the cellular poly-P-dependent ATP/GTP homeostasis, hence affecting the ability of *C. jejuni* to enter the VBNC state. Notably, it was shown that a putative ATP synthase was down regulated in the VBNC of *Enterococcus faecalis* (Heim et al., 2002), which prompted the speculation that the survival of VBNC under unfavorable conditions required alternative metabolic pathways to maintain energy (Heim et al., 2002). This possibly includes using energy that was stored as poly-P, further suggesting that an intact poly-P metabolism is essential for VBNC formation/persistence.

It was reported that poly-P occurs in starved and morphologically altered *V. parahaemolyticus* (Chen et al., 2009), and this molecule also accumulated in structurally intact coccoid forms of starved *H. pylori* (Nilsson et al., 2002). However, no direct mechanistic association between poly-P and the formation of VBNC has been described previously. Although further studies are needed to formulate a comprehensive mechanistic model of the involvement of poly-P and its enzymes in VBNC formation, the advances highlighted above might direct and facilitate future research into the VBNC state.

## THE FORMATE CONNECTION: FORMATE METABOLISM AND VBNC FORMATION IN *C. jejuni*

**FIGURE 1:**
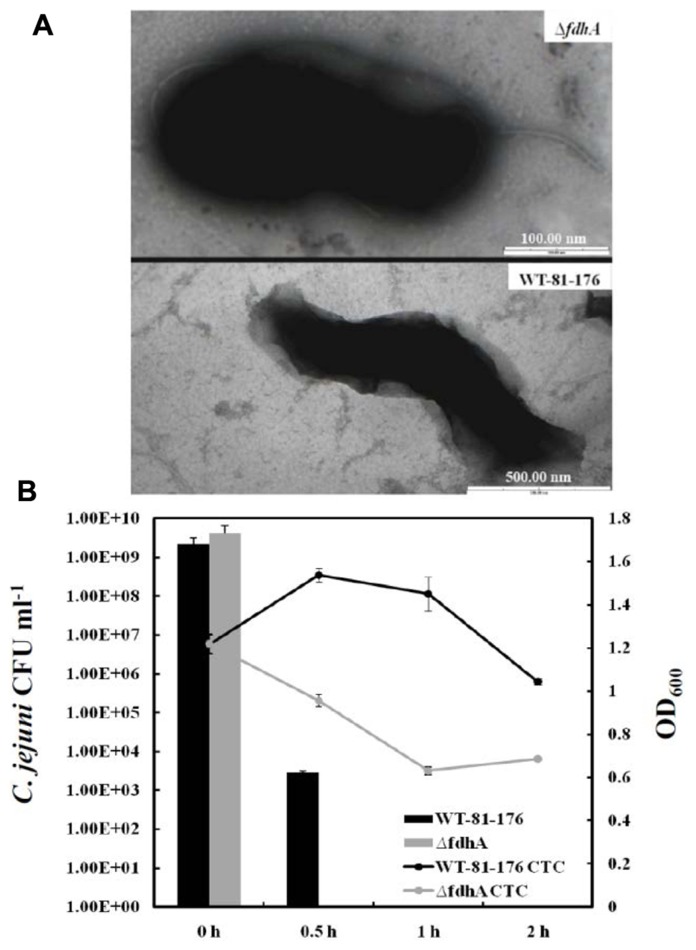
** (A)** Transmission electron microscopy images of the *C. jejuni* 81-176 wild-type strain and its formate dehydrogenase mutant (Δ*fdhA*) that is defective in the metabolism of formate. The Δ*fdhA* cells exhibited a “rounding up”/spherical morphology, which was also observed in our previous work analyzing the Δ*fdhA* of the *C. jejuni* NCTC-11168 wild-type strain (Kassem et al., 2012). **(B)** CFU enumerations and viability curves of the *C. jejuni* 81-176 wild-type and the Δ*fdhA* after exposure to formic acid (pH = 4.0; Kassem and Rajashekara, unpublished). The CFU were determined by plating on Mueller-Hinton agar, while the viability curve was constructed by staining with 5-cyano-2,3-ditolyl-tetrazolium chloride (CTC), which was used to determine the respiratory activity (hence viability) as described in Gangaiah et al. (2009) and elsewhere.

The findings linking poly-P to VBNC formation in *C. jejuni* will generate many extrapolations and questions. For example, the ATP/GTP pools in the cell are not only affected by poly-P and its enzymes; hence, could there be other factors impacting this pool and also contributing to VBNC formation in *C. jejuni*? Further, the Δ*ppk2* mutant only responded to formic acid with reduced VBNC formation as compared to the parental strain, however, there was no difference in the colony forming units (CFU) counts between the mutant and the wild-type after challenge with other organic acids (acetic acid and propionic acid) and hydrochloric acid (Gangaiah et al., 2010). Could the latter observation suggest some specific relationship between formic acid and VBNC formation in *C. jejuni*? As a matter of fact, the previous two questions are intimately linked, because *C. jejuni* possesses a highly branched respiratory chain that serves in energy production (Myers and Kelly, 2004), while one of the major energy sources for this bacterium is formate that is metabolized by the periplasmic respiratory protein, formate dehydrogenase (Hoffman and Goodman, 1982; Weerakoon et al., 2009). This is not surprising because formate is a byproduct of fermentation that occurs in the hosts’ gut, which is the preferable niche for *C. jejuni* (Weerakoon et al., 2009). It was also reported that the inactivation of the formate dehydrogenase (*fdh*) in this bacterium resulted in round shaped cells similar to those associated with the VBNC (**Figure 1A**); however, the *fdh* mutant did not lose culturability under normal growth conditions (Kassem et al., 2012). Further, when the *fdh* mutant was challenged with formic acid as described earlier, it showed a significant decrease in culturability and viability as compared to the wild-type, indicating a severe inability to form VBNC (unpublished data; **Figure 1B**). Therefore, it can be concluded that formate dehydrogenase and formate metabolism are associated with VBNC formation in *C. jejuni*, likely via their role in energy production. Along these lines, a question worthy of investigation is whether the energy produced by formate metabolism may be linked to poly-P accumulation/degradation? Regardless, both the formate and poly-P metabolism are associated with energy and VBNC production in *C. jejuni*, further confirming the role of energy conservation in VBNC formation and subsequent survival.

## COVETED POTENTIALS: CONCLUSIONS AND CLOSING REMARKS

It has been reported that poly-P and cognate enzymes occur in many bacterial species, suggesting that poly-P metabolism might be ubiquitous in the prokaryotes (Kornberg et al., 1999; Rao et al., 2009). Further, many of these species have a confirmed ability to enter the VBNC state (**Table 2**), which increases the appeal of a possible VBNC–poly-P link as a potential universal denominator in the formation of this cellular state. While the latter needs further experimental proof, poly-P appears to be a contributor to VBNC formation in the unconventional *C. jejuni*. Subsequently, the bearings of the findings listed above on efforts aiming at reducing *C. jejuni* in the food chain might be important, because it is already known that *C. jejuni* VBNC can adhere to edible products (e.g., on the skin of chicken carcasses; Jang et al., 2007). Further, phosphate-containing chemicals and weak organic acids (e.g., acetate and formate) have either been typically used or investigated as means to process, preserve, or decontaminate foods (Sofos and Smith, 1998; Capita et al., 2002; Hirshfield et al., 2003; Ricke, 2003). Organic acids, including formic acid, have also been used or tested as potential feed additives to reduce food-borne pathogens in animals including chickens, the primary reservoir of *C. jejuni* (Ricke, 2003). However, these chemicals might be inducers or modulators for the food-borne pathogen to enter the difficult-to-detect VBNC state.

**Table 2:** A list of food-borne bacterial pathogens that possess homologs of the PPK enzymes (Rao et al., 2009)

VBNC-forming food-borne bacteria	Polyphosphate kinase 1 (PPK1) and PPK2 homologs
*Campylobacter jejuni*	PPK1, PPK2
*Campylobacter coli*	PPK1, PPK2
*Enterococcus faecalis*	PPK1
*Escherichia coli*	PPK1, PPK2
*Helicobacter pylori*	PPK1
*Salmonella enterica *serovar, Enteritidis	PPK1
*S. *Typhimurium	PPK1
*Shigella dysenteriae* type 1	PPK1
*Shigella flexneri*	PPK1
*Vibrio cholerae*	PPK1, PPK2
*Vibrio parahaemolyticus*	PPK1, PPK2
*Yersinia enterocolitica*	PPK1

*These species/strains also have a documented ability to form VBNC (Rowan, 2004).
*

Admittedly, the role of VBNC in the spread of food-related infections is not clear; however, it can be argued that caution is warranted in scenarios that involve food safety. Therefore, investigations on VBNC regarding (a) factors that trigger their formation, (b) mechanisms of their formation, (c) their virulence properties, and (d) conditions that favor their infectivity are important for proper assessment of the impact of VBNC on food safety and public health.

## Conflict of Interest Statement

The authors declare that the research was conducted in the absence of any commercial or financial relationships that could be construed as a potential conflict of interest.
